# Enhancing the Conventional Culture: the Evaluation of Several Culture Media and Growth Conditions Improves the Isolation of Ruminal Bacteria

**DOI:** 10.1007/s00248-023-02319-2

**Published:** 2023-12-12

**Authors:** Lina Marcela Botero Rute, Alejandro Caro-Quintero, Alejandro Acosta-González

**Affiliations:** 1 AGROSAVIA, Km. 14 via Mosquera, Mosquera, Cundinamarca Colombia; 2 Maestría en Diseño y Gestión de Procesos, Facultad de Ingeniería, Universidad de la Sabana, Km. 7 Autopista Norte, Chia, 25001 Colombia; 3 Departamento de Biología, Facultad de Ciencias, Universidad Nacional de Colombia, Bogotá, Colombia; 4 Bioprospection Research Group (GIBP), Facultad de Ingeniería, Universidad de La Sabana, Km. 7 Autopista Norte, Chia, 25001 Colombia

**Keywords:** Anaerobic bacteria, Culturomics, Rumen microbiome, Metataxonomy

## Abstract

The rumen microbiota is critical in cattle digestion. Still, its low cultivability makes it difficult to study its ecological function and biotechnological potential. To improve the recovery of ruminal microorganisms, this study combined the evaluation of several cultivation parameters with metabarcoding analysis. The parameters tested comprised eight media cultures, three sample dilutions (10^−2^, 10^−6^, 10^−12^), and two incubation times (3 and 7 days). Bacterial populations were determined through Illumina sequencing of 16S rRNA from three biological replicates. The results indicate that none of the culture media recovered all rumen populations and that there was an altered relative abundance of the dominant phyla. In the rumen, Bacteroidetes and Firmicutes comprised 75% and 15% of the relative abundance, respectively, while in the culture media, these were 15% and 60%, respectively. Principal coordinate analysis (PCoA) of the bacterial community revealed significant shifts in population composition due to dilution, with 10^−2^ and 10^−6^ dilutions clustered closely while the 10^−12^ dilution differed markedly. In contrast, incubation duration did not influence population diversity. According to the results, two media, CAN and KNT, were selected based on their ability to recover more similar populations compared to the rumen sample. The metataxonomic study showed that CAN media had consistent reproducibility over time, while KNT showed enrichment of different taxa due to the use of rumen fluid as a substrate. From these, 64 pure cultures were obtained and 54 were identified through 16S rRNA gene sequencing. Being *Streptococcus* the most frequently isolated genus, this prevalence contrasts with the liquid media composition, underscoring the importance of refining single colony isolation strategies. Although no culture medium could replicate the native rumen bacterial population perfectly, our findings highlight the potential of CAN and KNT media in recovering populations that are more closely aligned to natural rumen conditions. In conclusion, our study emphasizes the importance of integrating molecular approaches in selecting suitable cultivation media and parameters to depict rumen bacteria accurately.

**Supplementary Information:**

The online version contains supplementary material available at 10.1007/s00248-023-02319-2.

## Introduction

The rumen, the first compartment of the ruminants’ digestive tract, is a fermentation chamber that hosts a diverse and dynamic microbial community referred to as the rumen microbiota [1, 2]. This community plays a crucial role in the digestive processes and nutrient utilization of ruminants and is essential for their health and productivity. The interactions among the bacteria, archaea, fungi, ciliate protozoa, and viruses form a complex network that helps maintain a stable rumen environment [3–7].

The manipulation of rumen microbial composition and functionality has been the subject of extensive investigation, especially concerning the advancement of dietary supplements, probiotics, chemical substances, and feeding regimens. These endeavors aim to enhance animal productivity and health while concurrently mitigating environmental consequences, such as methane emission [8–13]. Despite its significance, only a small fraction has been isolated and characterized, hindering the efforts towards more sustainable agriculture [14–16].

Studies indicate that the rumen microbiota is dominated by bacteria, particularly those belonging to the phylum Firmicutes and Bacteroidetes, families such as *Lachnospiraceae*, *Ruminococcaceae*, *Bacteroidales*, and *Clostridiales*, and the genera *Prevotella, Butyrivibrio*, and *Ruminococcus* [3, 17–19]. Most of what is known about these bacteria has been obtained through cultivation-dependent techniques, which have allowed for their physiological characterization. Despite over 150 years of cultivation efforts, merely 800 new bacterial species are identified annually representing only a fraction of the estimated global bacterial diversity [16, 20–22].

Traditional culture-dependent methods, such as the roll tube technique and the use of dilutions and ruminal fluid–based media, have been used to isolate ruminal microorganisms [23, 24]. However, these methods are limited as they only encompass 10–20% of the ruminal population. Although numerous efforts have been made to isolate ruminal microorganisms, only 3.6% (61/1698 OTUs) of the OTUs reported by sequencing have cultivated representatives, and only 117 bacterial species of the rumen are found in reference culture collections [15, 25, 26]. These findings highlight the need for continued efforts to isolate and characterize the rumen microbiota using a combination of traditional and innovative techniques [25].

In this study, we have undertaken a comprehensive approach to enhance the cultivation of ruminal bacteria utilizing a hybrid strategy that combines classical microbiology techniques with metabarcoding methods. Our approach consists of two main phases. In the initial phase, we employed metataxonomy to assess how various culture media and growth conditions impact the enrichment of ruminal anaerobic bacteria. During the subsequent phase, we leveraged this optimized cultivation approach to isolate bacteria, identify them, and then compare this cultivated diversity to the metabarcoding findings from the first phase.

## Materials and Methods

### Sampling and Animal Preparation

The samples were collected from a fistulated male Holstein bovine belonging to AGROSAVIA C.I, Tibaitatá (4°41′43.5″N 74°12′19.8″W, elevation 2516 m). The animal was kept at the Tibaitatá Research Center and allowed to feed freely on Kikuyu grass (*Pennisetum clandestinum*) forage and water diet condition for a week; the animal was induced to a fasting condition for 16 h to increase the probability of obtaining a higher number of species in the rumen liquor. The animal was allowed to feed and ruminal samples were taken 1 h later. Three ruminal fluid samples (technical replicates) were collected through the fistula in tubes previously gasified with a CO_2_ mixture to N_2_ ratio (80:20), filling the tubes to the top. Then, the fluid was inoculated in tubes with dilution media [27], using an Atmosbag glove bag (Merck KGaA, Darmstadt, Germany) with CO_2_ to N_2_ ratio (80:20) atmosphere. The inoculated tubes were maintained at a constant temperature of 40 °C to maintain anaerobic ruminal conditions until being processed in the laboratory (less than 30 min). The remaining collected fluid was frozen at −20 °C with glycerol 15% as cryoprotectant.

### Culture Media Preparation

For the culture of ruminal microorganisms, 10-mL tubes of eight anaerobic media were prepared, named ER medium (ER), CAN medium (CAN) [28], glucose/cellobiose medium (GC) [27], Goodman medium (GOOD) [29], Kenters medium (KNT) [30], Nyonyo medium (NYO) [31], Kikuyu medium (KYO), and Red clover medium (TRB), these two designed by the laboratory group (media composition in supplementary material SM Tables 1–8). All the media were prepared under anoxic conditions and were supplemented with resazurin sodium salt (Merck KGaA, Darmstadt, Germany) to guarantee the anaerobic atmosphere.

### Culture Conditions for the Enrichment of Rumen Microorganisms

The three samples collected previously were diluted in a dilution medium (media composition in supplementary material SM Table 9). Serial dilutions 1/10 in a volume of 9 mL were done up to 10^−12^ dilutions. Three dilutions were selected (10^−2^, 10^−6^, 10^−12^) to be used as inoculum, and 1 mL of each dilution was inoculated in each culture media evaluation. For each medium, we assessed the effect of incubation at 3 and 7 days. All treatments were evaluated in triplicates with three replicates and 147 samples were taken. All media were incubated at 39 °C ± 2 °C without stirring.

The AGROSAVIA committee approved the animal handling following the “Format for the Use of Animals” (AGROSAVIA) and law 84 of 1989 of The Congress of the Republic of Colombia. The sampling was carried out to ensure animal welfare, according to the internal regulations of the corporation and the Colombian legislation mentioned above.

### Meta-taxonomic Methods to Characterize Recovering Microbial Communities

#### DNA Extraction

DNA extraction was done for each medium after the corresponding incubation time. The cells were concentrated by centrifugation (Thermo Scientific™ Sorvall™ Legend™ XT/XF, DE, USA) at 13,000 rpm for 10 min at 4 °C, discarding the supernatant and resuspended the cells in 2 mL of 0.85% sterile saline solution. The volume was distributed equally in cryovials, leaving one of them as a counter sample. DNA extraction was performed by phenol to chloroform ratio [32]. DNA concentration was quantified using a NanoDrop™ 2000/2000c Spectrophotometer (Thermo Fisher Scientific, DE, USA), while the DNA quality was determined by gel electrophoresis with a 1.5% agarose gel (w/v) and with the absorbance ratio obtained at 260/230 nm and 260/280 nm. The DNA concentration was adjusted to 20 μg/μL.

#### Bacterial Metabarcoding using 16S rRNA Gene

The V3–V4 region of the 16S rRNA was amplified using primers 515F-806R (515F-5′-GTGCCAGCMGCCGCGG-3′; 806R-5′-GGACTACHVGGGTWTCTAAT-3′) with an adapter sequence in the 5′region. Subsequently, barcoding primers with a 10-bp sequence complementary to the adapter were used, generating an amplicon with different barcoding in each sample [33].

#### Preparation of Amplicon Library

The first amplification and subsequent addition of the “barcode” were performed by two consecutive PCRs. The first PCR reaction was performed in triplicate for each sample. The reaction contained 0.1 μL of Platinum™ Taq DNA Polymerase High Fidelity (Invitrogen Carlsbad, CA, USA), 0.75 μL MgCl2 50 mM, 2.5 μL Buffer-Mg 10X, 0.5 μL dNTPs 10 mM, 0.5 μL (10 μM) of each of the primers, 2 μL DNA, and 18.15 μL of distilled water ultrapure (Invitrogen Carlsbad, CA, USA), under the following conditions: (i) 94 °C for 3 min, (ii) 35 cycles at 94 °C for 45 s, 50 °C for 60 s, and 72 °C for 90 s, and (iii) a final extension at 72 °C for 10 min (T100™ Thermal Cycler, BioRad, CA, USA). The amplifications were verified on 1.5% agarose gels. The triplicates were purified with AMPure XP beads (Beckman Coulter, Inc. IN, USA) and combined in a single sample.

Afterward, the second PCR was performed with the combined and purified product of the first reaction. For this reaction, 5 μL of the purified product was used as a template, one μL (10 μM) of each barcoding primer, 0.1 μL of Platinum™ Taq DNA Polymerase High Fidelity (Invitrogen Carlsbad, CA, USA), Taq Platinum (Invitrogen), 0.75 μL MgCl2 50 mM, 2.5 μL Buffer-Mg 10X, 0.5 μL dNTPs 10 mM, and 14.15 μL of ultrapure distilled water (Invitrogen, Carlsbad, CA, USA). PCR was performed using the conditions described above for the first reaction. Nevertheless, the number of cycles was completed with 12 cycles. The final PCR products were verified on 1.5% agarose gels and purified with AMPure XP beads (Beckman Coulter, Brea, CA); amplified products were quantified on a Qubit 2.0 Fluorometer (Invitrogen Carlsbad, CA, USA). The libraries were adjusted to the requirements of the MiSeq system of the Illumina® platform [34, 35]. The purified amplicons were pooled in equimolar concentrations and pair-end sequenced (250 nt PE reads) on an Illumina MiSeq at the Microbial genomics laboratory of the Molecular Genetics and Antimicrobial Resistance Unit at Universidad El Bosque, Bogotá, Colombia.

#### Analysis of 16S rRNA Metabarcoding

The analyses for sequenced samples were analyzed using the Qiime2 software (version 2019.7) [36]. The analysis was done with the following steps: quality control using the DADA2 command [37], allowing to filter sequences, eliminate chimeric sequences, and set the trim and trunk parameters; --p-trunc-len-f 270 --p-trunc-len-r 220 --p-trim-left-f 10 --p-trim-left-r 10, allowing to preserve sequences with high quality; determination of alpha and beta diversity indices and their visualization by principal coordinate analysis (PCoA) for beta diversity; final stage: taxonomic study of the samples and assignment of OTUS (operational taxonomic units) using as reference classifier, the 16S rRNA Greengenes database (http://greengenes.lbl.gov), the percentage of 97% was used as the minimum similarity parameter for taxonomic assignment. Alpha- and beta-diversity analyses and taxonomic analyses of relative abundance were performed in RStudio (v.3.5.3), using the Qiime2R package. The distributions of distances obtained in beta diversity were determined with the UniFrac.

### Cultivation Strategy for the Isolation of Microorganisms

Rumen fluid was obtained following the guidelines in the “Sampling and Animal Preparation” section, with three technical replicates collected. Our isolation strategy was based on two critical criteria: the simplicity of media preparation and the enrichment of microorganisms with low abundance. Accordingly, CAN and KNT media were selected to isolate the ruminal bacteria. In brief, the rumen fluid samples were serially diluted to a 10^−12^ concentration; 1 mL of this dilution was used as inoculum in tubes with 9 mL of media and were incubated at 39 °C for 3 and 7 days. After incubation, each tube underwent further dilutions, and 0.5 mL of these dilutions was combined with 4.5 mL of liquid media. This 5-mL mixture was then spread across the tube’s surface and rolled to establish an agar layer [23]. Incubation of the roll tubes was initiated when colonies became visible, and individual colonies were isolated and transferred to liquid media based on the origin of each tube. The purity of colonies was verified using phase contrast microscopy. Cultures with multiple morphologies were subject again to the roll tube method. Pure isolates were stored in a secondary tube within an anaerobic dilution media (SM Table 9) containing 15% (v/v) glycerol, ensuring the medium’s reducing conditions were maintained. The process was conducted under anaerobic conditions in a COY chamber at 39 °C ± 2 °C.

### Molecular Characterization of the Recovered Isolates

For the molecular identification of the isolates, DNA extractions were performed by the phenol to chloroform ratio method described in the “DNA Extraction” section. The amplification of the 16S rRNA gene was performed using the 27f (5′-AGAGTTTGATCMTGGCTCAG-3′) and 1492r (5′-CGGTTACCTTGTTACGACTT-3′) universal primers. PCR was performed at a volume of 25 μL. The reaction consisted of 2.5 mL of 10 × buffer, 1 mL of 50 mM MgSO4, 0.5 mL of 10 mM DNTP mix (2.5 mM each, Invitrogen, Carlsbad, CA), 0.5 mL of each primer, 0.1 mL of Taq Polymerase Platinum High-Fidelity (Invitrogen, Carlsbad, CA), and 2 mL of DNA. The PCR program consisted of 2 min at 94 °C followed by 10 cycles of 15 s at 94 °C, 30 s at 46 °C, and 1 min at 72 °C, followed by 20 cycles of 15 s at 94 °C, 30 s at 50 °C, and 2 min at 72 °C, with a final extension of 7 min at 72 °C. The amplified products were visualized by electrophoresis using 2 μL of the amplified PCR product on a 1.5% (w/v) gel. PCR products were sequenced at Corpogen (Bogotá., Colombia).

### Phylogenetic Analyses

The obtained sequences were quality checked using the Geneious Prime program (V.2019.2.1) and identified through the BLASTN tool provided by the NCBI (https://blast.ncbi.nlm.nih.gov/). A phylogenetic analysis was performed using MEGA 7.0.26 with the ClustalW tool, with 16S rRNA gene sequences aligned against references obtained from GenBank. An evolutionary distance tree was constructed using the neighbor-joining distance method and the Kimura 2P model, with a bootstrap of 1000 iterations and *Methanobrevibacter ruminantium* (GenBank NR_042784.1) used as an outgroup.

### Determination of Microbial Structure by Electron Microscopy (SEM)

Scanning electron microscopy (SEM) was conducted using an LYRA3 TESCAN ion beam microscope at the Microscopy Center of the Universidad de Los Andes for morphological description of the isolates. Samples were taken from pure cultures by centrifuging 1 mL of the culture at 13,000 rpm for 10 min, after which the supernatant was removed, and the cells resuspended in a sterile 0.85% saline solution. The cells were then fixed with 2.5% glutaraldehyde solution for 12 h and centrifuged at 10,000 rpm for 5 min, after which the glutaraldehyde was removed. The cells were washed with molecular grade water to remove impurities. They were then subjected to a series of ethanol washes, starting with 70% ethanol for 5 min and followed by two washes with 95% ethanol for 10 min each. Finally, the cells were washed thrice with 100% ethanol for 20 min each, centrifugation at 10,000 rpm for 3 min after each wash, and ethanol addition. Following fixation and dehydration, the samples were processed and photographed.

### Taxonomic Comparison Analysis of the Enrichment and Isolates Obtained in Pure Culture

As previously described, a new metataxonomic library was prepared, using DNA extracted from the media detailed in the “Cultivation Strategy for the Isolation of Microorganisms” section. To assess taxonomic enrichment reproducibility, this library was taxonomically compared with those from the initial media evaluation. Furthermore, comparisons were made with taxonomic groups derived from single colony isolation. This was done to estimate how many groups were successfully recovered and identify any potential losses during the transfer from the liquid media to the rolling tube agar.

## Results

### Bacterial Diversity and Structure

**Fig. 1:**
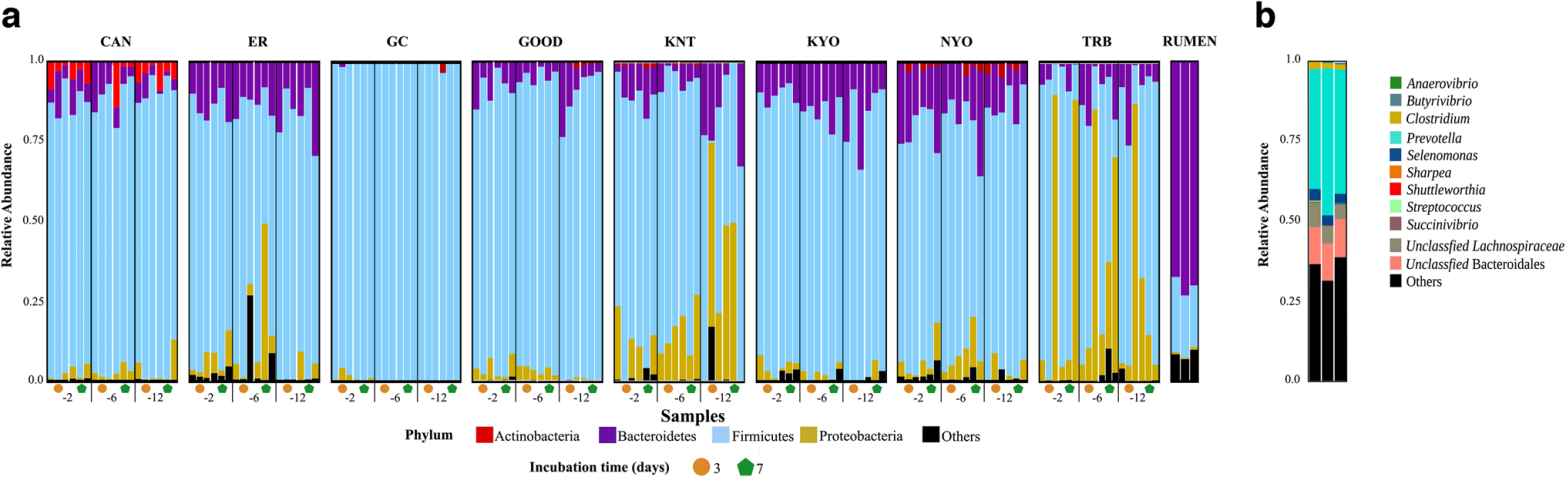
Comparative analysis of microbial composition. **a** The relative abundance of predominant phyla in various media is shown. Below, treatment groups are categorized by dilutions: -2 (10^-2^), -6 (10^-6^), and -12 (10^-12^). Orange and green symbols denote treatments incubated for 3 and 7 days. **b** The panel presents the relative abundance of the most prevalent genera in rumen fluid

A total of 147 culture samples were sequenced, comprising 48 treatments with three biological replicates each, along with three rumen samples. Additionally, a rumen sample was used as a reference to gauge the original diversity. From this, 9,827,732 reads were generated, averaging 39,538 reads per sample. After undergoing quality control filtering, 5,733,051 reads were retained, each averaging 272 bp in length. Clustering of these reads yielded 3764 assigned operational taxonomic units (OTUs). Upon taxonomic classification, 14 phyla were identified as follows: Acidobacteria, Actinobacteria, Bacteroidetes, Elusimicrobia, Fibrobacteres, Firmicutes, Fusobacteria, Lentisphaerae, Planctomycetes, Proteobacteria, Spirochaetes, Synergistetes, Tenericutes, and Verrucomicrobia. However, only four phyla exhibited a relative abundance exceeding 1 The remaining phyla were collectively categorized as “Others.” Among all the treatments, Firmicutes dominated with a 79% abundance, followed by Bacteroidetes at 11%, Proteobacteria at 8%, and Actinobacteria at 1%. Bacteroidetes were predominant at 72%, in the rumen fluid, while Firmicutes followed at 20%. All media was observed to have influenced the phyla composition, with the GC medium having over 90% Firmicutes across all treatments (Fig. 1).

The composition was determined at the genus level for each sample, including the rumen fluid samples. Eleven genera were abundant across the media and treatments: *Acidaminococcus*, *Anaerovibrio*, *Butyrivibrio*, *Clostridium*, *Olsenella*, *Prevotella*, *Selenomonas*, *Sharpea*, *Shuttleworthia*, *Streptococcus*, and *Succinivibrio*, as well as two family groups, Enterobacteriaceae and Lachnospiraceae, and the Bacteroidales order. Only genera with a relative abundance above 1% were considered, with those with lower abundance being grouped as “Others.” In contrast, the genus level diversity of the rumen sample showed the dominance of *Prevotella* (45%), followed by a group of the Bacteroidales order (11%) and the *Lachnospiraceae* family (6%) (Fig. 1).

### Bacterial Taxonomic Composition Across Various Media and Dilutions

**Fig. 2:**
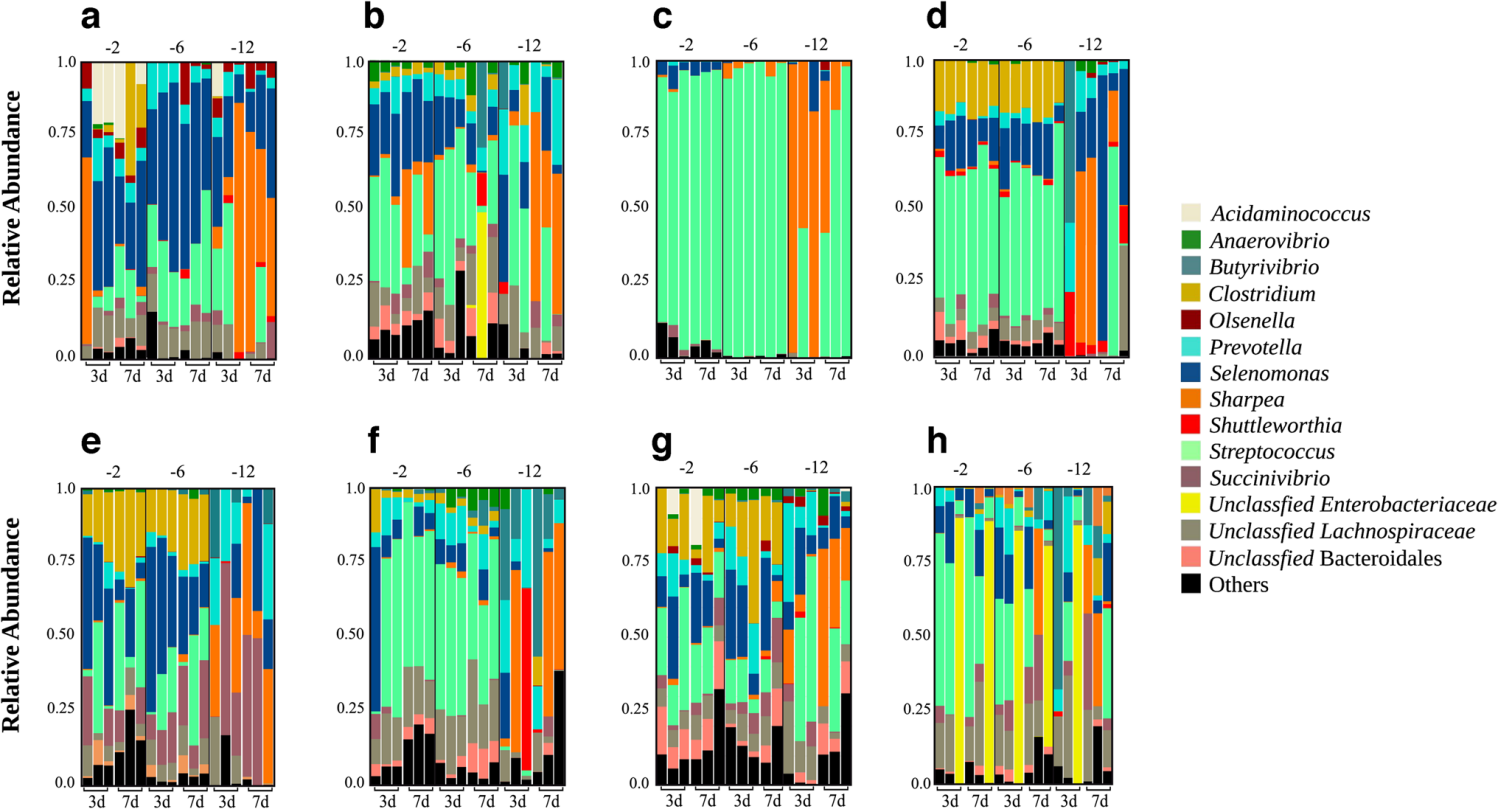
Abundance of dominant genera in evaluated culture media across various dilutions and incubation times. Various dilutions and incubation periods show the relative abundance of key genera within different media. The media are sequentially organized from left to right. On top: **a** CAN, **b** ER, **c** GC, and **d** GOOD. Bottom: **e** KNT, **f** KYO, **g** NYO, and **h** TRB media

The analysis of the bacteria taxonomic composition shows that the composition and richness obtained for the 10^−2^ and 10^−6^ dilutions were similar, within each media. In contrast, the media inoculated with the 10^−12^ had the lower richness values. Some genera were enriched in almost all evaluated treatments, with *Selenomonas* being highly enriched in all cases as well as *Streptococcus* (Fig. 2c). However, *Streptococcus* appears to be less abundant in the presence of *Sharpea*, which is only abundant in the 10^−12^ samples. There is no abundance of *Streptococcus* at these dilutions in the KNT (Fig. 2e) and KYO (Fig. 2f) media. *Prevotella*, one of the most abundant genera, is present in seven of the eight media, with its abundance being different in each medium, but with the ER (Fig. 2b) and KYO (Fig. 2f) media showing a higher abundance of the genus. The Lachnospiraceae family and Bacteroidales order are enriched in all media, except GC. Other taxa are only present in a few media, such as *Olsenella*, which belongs to the Actinobacteria phylum and shows abundance in the CAN (Fig. 2a) and NYO (Fig. 2g) media. Succinivibrio, on the other hand, is present in all dilutions with an increase at higher dilutions, reaching 50% abundance in the 10^−12^ samples (Fig. 2e), while the highest abundance of Clostridium is observed in the GOOD, KNT, and NYO media, with an average of 20% abundance in the 10^−2^ to 10^−6^ dilutions.

### Alfa Diversity Analysis

The Shannon index was used as a measure of sample diversity. The rumen had the highest richness, with the media being grouped into three categories based on their richness: the NYO and ER media had the highest richness [4–6], followed by the CAN, KYO, KNT, and GOOD media with a medium richness [3, 4], and the GC and TRB media had the lowest richness according to the index [1–3] (Fig. 1SM). The 10^−2^ dilution had the highest richness index, which decreased with increasing dilution. The GC and TRB media showed a different dilution pattern, with a similar richness index at the lowest and highest dilution (Fig. 1SM).

### Effect of Parameters on Bacterial Community Composition, a Multivariate Analysis

**Fig. 3:**
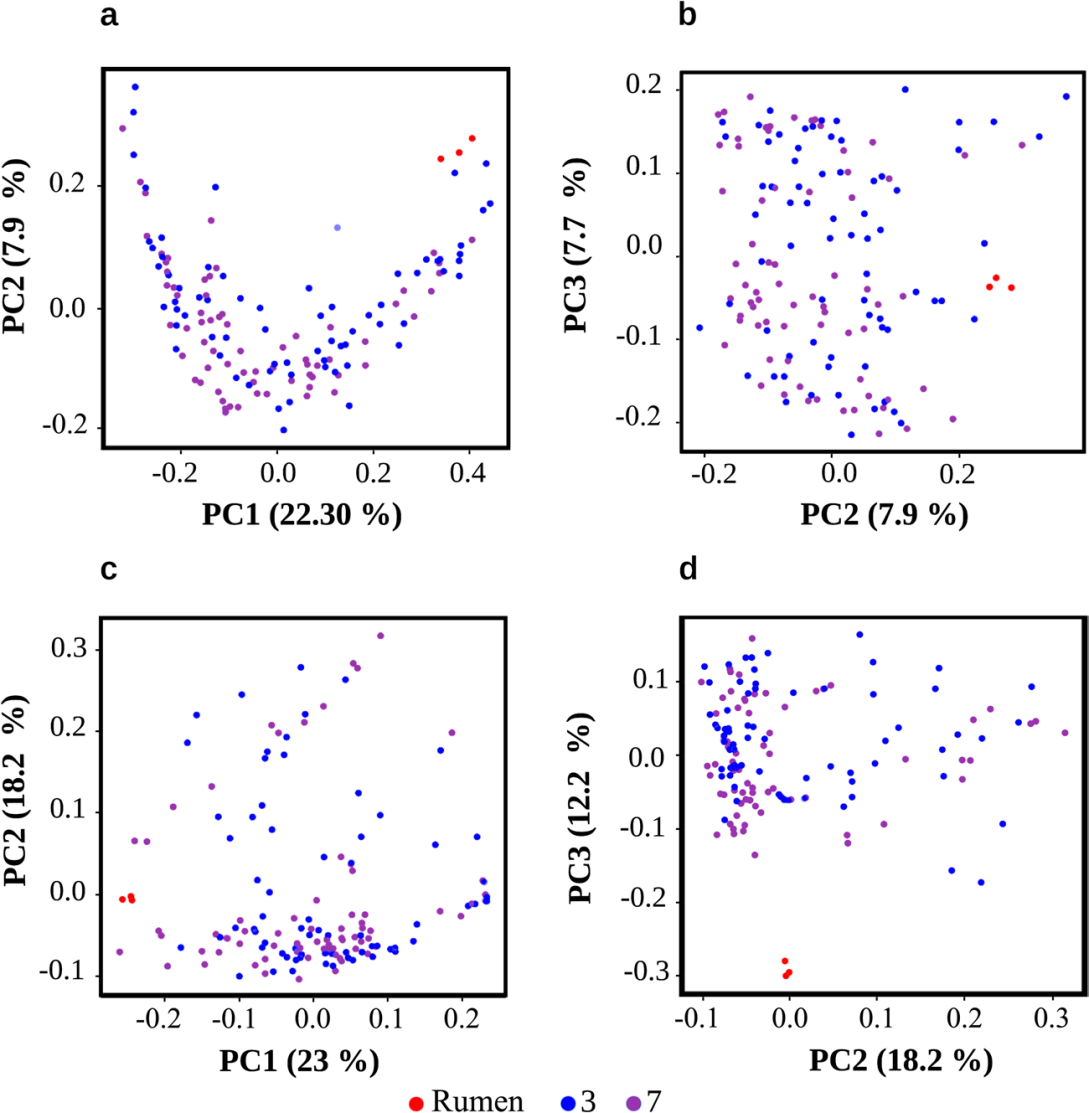
Principal Coordinate Analysis (PCoA) of samples categorized by incubation time. On top: **a**, **b** PCoA plots using unweighted UniFrac distances. Bottom: **c**, **d** PCoA plots using the weighted UniFrac distance. The first three principal components (PC) are shown for both distances. The red dots represent rumen samples, blue indicates samples at 3 days of incubation, and purple denotes samples at 7 days of incubation

The effect of the variables on the community was determined using the Unifrac index (beta diversity). The PCoA (Fig. 3) shows the impact of incubation days and their relationship with the rumen; the data for both days are heterogeneously distributed, with this behavior observed in both PCoA configurations, with the PC1 vs PC2 and PC2 vs PC3 axes, for both the unweighted (Fig. 3a, b) and weighted (Fig. 3c, d) data.

**Fig. 4:**
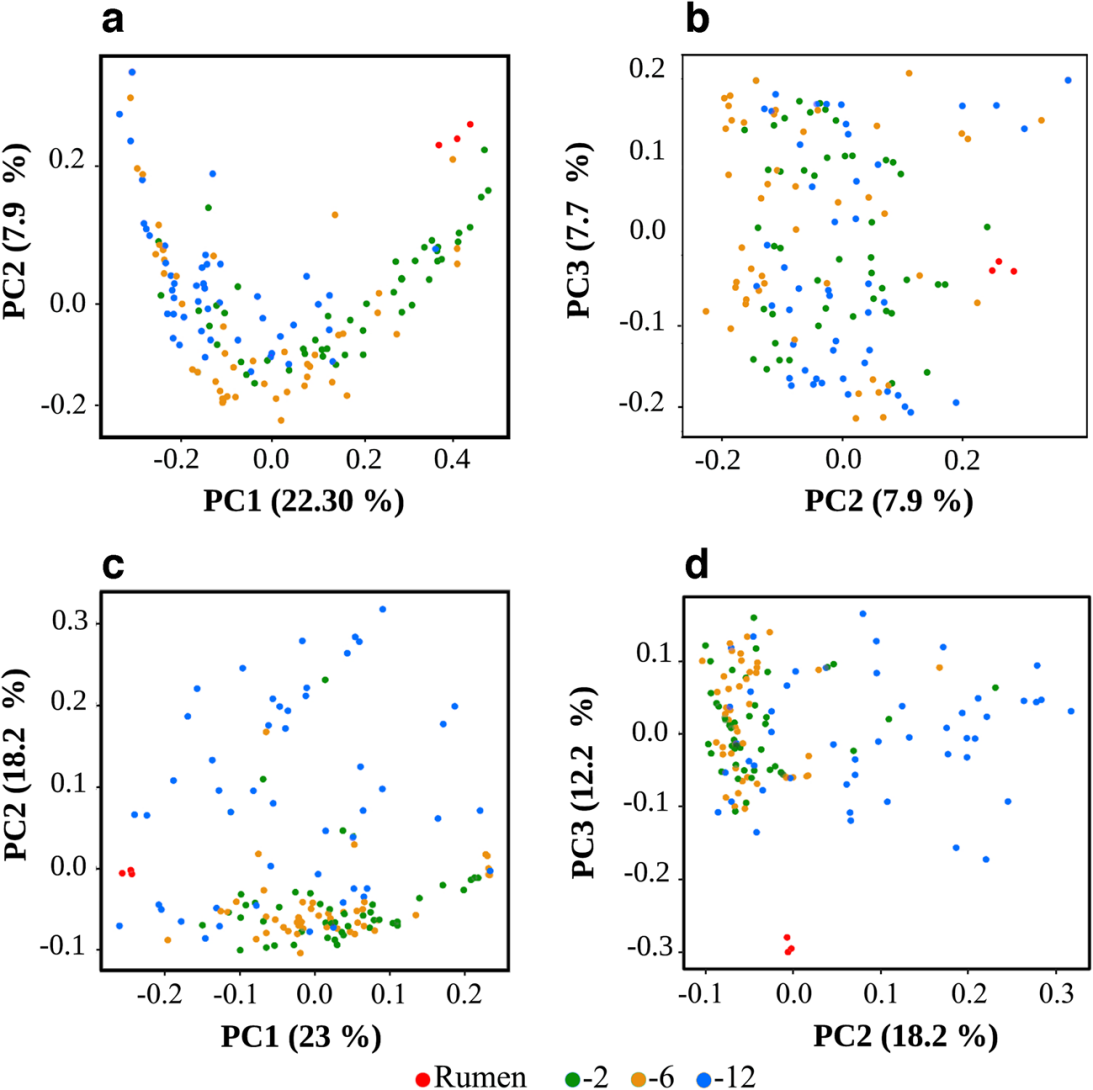
Principal coordinate analysis (PCoA) of samples categorized by dilution. The cluster analysis for the dilution variable is shown using the Unifrac metric. On top: **a**, **b** PCoA plots based on unweighted UniFrac distances. Bottom: **c**, **d** PCoA plots using weighted UniFrac distances. The first three principal components (PC) are shown for both distances. Red dots represent rumen samples, green for data with a 10^-2^ dilution, yellow for data with a 10^-6^ dilution, and blue for data with a 10^-12^ dilution

Two main clusters are observed for the effect of dilution on the community. One cluster is composed of the 10^−2^ and 10^−6^ dilutions, with some data overlap. The second cluster comprises the 10^−12^ dilution, with all the data being distant from cluster one and the rumen. The effect of dilution on the community is observed by the formation of data clusters, which are determined by the type of dilution. The phylogenetic diversity (unweighted) of the samples (Fig. 4a) establishes three patterns of a succession of data concerning the rumen: a group located close to the rumen being the 10^−2^ dilution, followed by the 10^−6^ dilution at a medium distance, and the most distant group being the 10^−12^ dilution; this behavior is not distinguishable when using the PC2 vs PC3 axes (Fig. 4b). The weighted data (Fig. 4c, d) shows a cluster of data belonging to the 10^−2^ and 10^−6^ dilutions, suggesting that the relative abundance of these samples is similar, while moving away from the highest dilution (10^−12^), where it indicates a change in both population and abundance. This behavior is supported by the changes observed at the taxonomic level for each of the dilution (Fig. 2). Also, some data from the 10^−2^ dilution in the PCoA (Fig. 4a) suggest a close relationship with the rumen samples.

### Taxonomic Identification of Isolates

The previous results have demonstrated the enrichment of essential microorganisms, including *Butyrivibrio*, *Olsenella*, *Prevotella*, *Sharpea*, *Selenomonas*, *Succinivibrio*, *Streptococcus*, and *Shuttleworthia* in the highest dilution. While the incubation time did not significantly impact the microbial community, it was considered during the isolation procedure. Sixty pure colonies were obtained and characterized through gram staining and morphology, with 41 classified as gram-positive, 14 as gram-negative, and 5 as gram-variable. Morphological observations of these colonies revealed the presence of both rod-shaped and coccoid cells. Ten genera and 12 species related to them were identified through the 16S rRNA gene. *Actinomyces ruminicola*, *Butyrivibrio* sp., *Limosilactobacillus mucosae*, *Oribacterium* sp., *Pediococcus acidilactici*, *Pseudobutyrivibrio* sp., *Selenomonas ruminantium*, *Staphylococcus epidermidis*, *Staphylococcus warneri*, *Staphylococcus pasteuri*, *Streptococcus orisasini*, *Streptococcus equinus*, *Streptococcus lutetiensis*, *Streptococcus salivarius*, and *Succinivibrio dextrinosolvens*. Table 1 shows the results for each isolate and their similarity percentage using the NCBI database, and four isolates were not identified for the low sequence quality.

**Table 1:** Taxonomic assignment of isolated bacteria. Closest relatives were identified by comparison of 16S rRNA gene sequences against the NCBI 16S rRNA database the percentage of identity was obtained from the BLAST results

Isolate	Medium	Incubation time (days)	% id	Genus assignment	Closest specie identity
i1	CAN	7	100	*Staphylococcus*	*warneri*
i2	CAN	7	100	*Staphylococcus*	*warneri*
i3	CAN	7	99	*Staphylococcus*	*pasteuri*
i4	CAN	7	100	*Staphylococcus*	*epidermidis*
i5	CAN	7	99	*Streptococcus*	*salivarius*
i6	KNT	7	94	*Pseudobutyrivibrio*	-
i7	KNT	7	94	*Pseudobutyrivibrio*	-
i8	KNT	3	100	*Streptococcus*	*equinus/lutetiensis*
i9	KNT	3	100	*Streptococcus*	*equinus/lutetiensis*
i10	KNT	3	100	*Streptococcus*	*equinus/lutetiensis*
i11	KNT	3	100	*Streptococcus*	*equinus/lutetiensis*
i12	CAN	7	*-*	*Low quality*	
i13	CAN	7	100	*Pediococcus*	*acidilactici*
i14	KNT	3	99	*Streptococcus*	*equinus*
i15	CAN	7	99	*Staphylococcus*	*epidermidis*
i16	CAN	7	100	*Streptococcus*	*salivarius*
i17	CAN	7	99	*Staphylococcus*	*epidermidis*
i18	CAN	7	99	*Staphylococcus*	*epidermidis*
i19	CAN	7	99	*Staphylococcus*	*epidermidis*
i20	KNT	3		*Selenomonas*	*ruminantium*
i21	KNT	7	100	*Staphylococcus*	*warneri*
i22	KNT	3	100	*Streptococcus*	*equinus/lutetiensis*
i23	KNT	7	99	*Streptococcus*	*lutetiensis/equinus*
i24	KNT	7	99	*Streptococcus*	*lutetiensis/equinus*
i25	KNT	7	100	*Staphylococcus*	*warneri*
i26	KNT	7	100	*Streptococcus*	*lutetiensis/equinus*
i27	KNT	3	100	*Succinivibrio*	*dextrinosolvens*
i28	KNT	3	100	*Succinivibrio*	*dextrinosolvens*
i29	CAN	7	100	*Staphylococcus*	*warneri*
i30	CAN	7	99	*Streptococcus*	*orisasini*
i31	CAN	3	100	*Staphylococcus*	*warneri*
i32	KNT	7	*-*	*Low quality*	
i33	CAN	3	100	*Limosilactobacillus*	*mucosae*
i34	CAN	3	100	*Staphylococcus*	*pasteuri/warneri*
i35	CAN	3	88	*Selenomonas*	*ruminantium*
i36	KNT	3	98	*Oribacterium*	-
i37	KNT	7	99	*Streptococcus*	*lutetiensis/equinus*
i38	KNT	7	100	*Streptococcus*	*lutetiensis/equinus*
i39	CAN	3	100	*Limosilactobacillus*	*mucosae*
i40	KNT	7	*-*	*Low quality*	
i41	KNT	7	100	*Staphylococcus*	*warneri*
i42	KNT	3	98	*Oribacterium*	-
i43	KNT	7	100	*Succinivibrio*	*dextrinosolvens*
i44	KNT	7	*-*	*Low quality*	
i45	CAN	3	99	*Staphylococcus*	*pasteuri*
i46	KNT	3	99	*Succinivibrio*	*dextrinosolvens*
i47	KNT	7	99	*Succinivibrio*	*dextrinosolvens*
i48	KNT	7	99	*Succinivibrio*	*dextrinosolvens*
i49	CAN	3	99	*Selenomonas*	*ruminantium*
i50	KNT	7	100	*Succinivibrio*	*dextrinosolvens*
i51	KNT	7	99	*Succinivibrio*	*dextrinosolvens*
i52	KNT	7	99	*Succinivibrio*	*dextrinosolvens*
i53	KNT	7	100	*Succinivibrio*	*dextrinosolvens*
i54	KNT	7	100	*Succinivibrio*	*dextrinosolvens*
i55	KNT	7	100	*Succinivibrio*	*dextrinosolvens*
i56	KNT	7	98	*Actinomyces*	*ruminicola*
i57	KNT	3	100	*Butyrivibrio*	*fibrisolvens*
	KNT	3	99	*Butyrivibrio*	*hungatei*
i58	KNT	7	98	*Actinomyces*	*ruminicola*
i59	CAN	7	77	*Streptococcus*	*orisasini*
i60	KNT	3	100	*Butyrivibrio*	*fibrisolvens*

*CAN* CAN media, *KNT* Kenter’s media

**Fig. 5:**
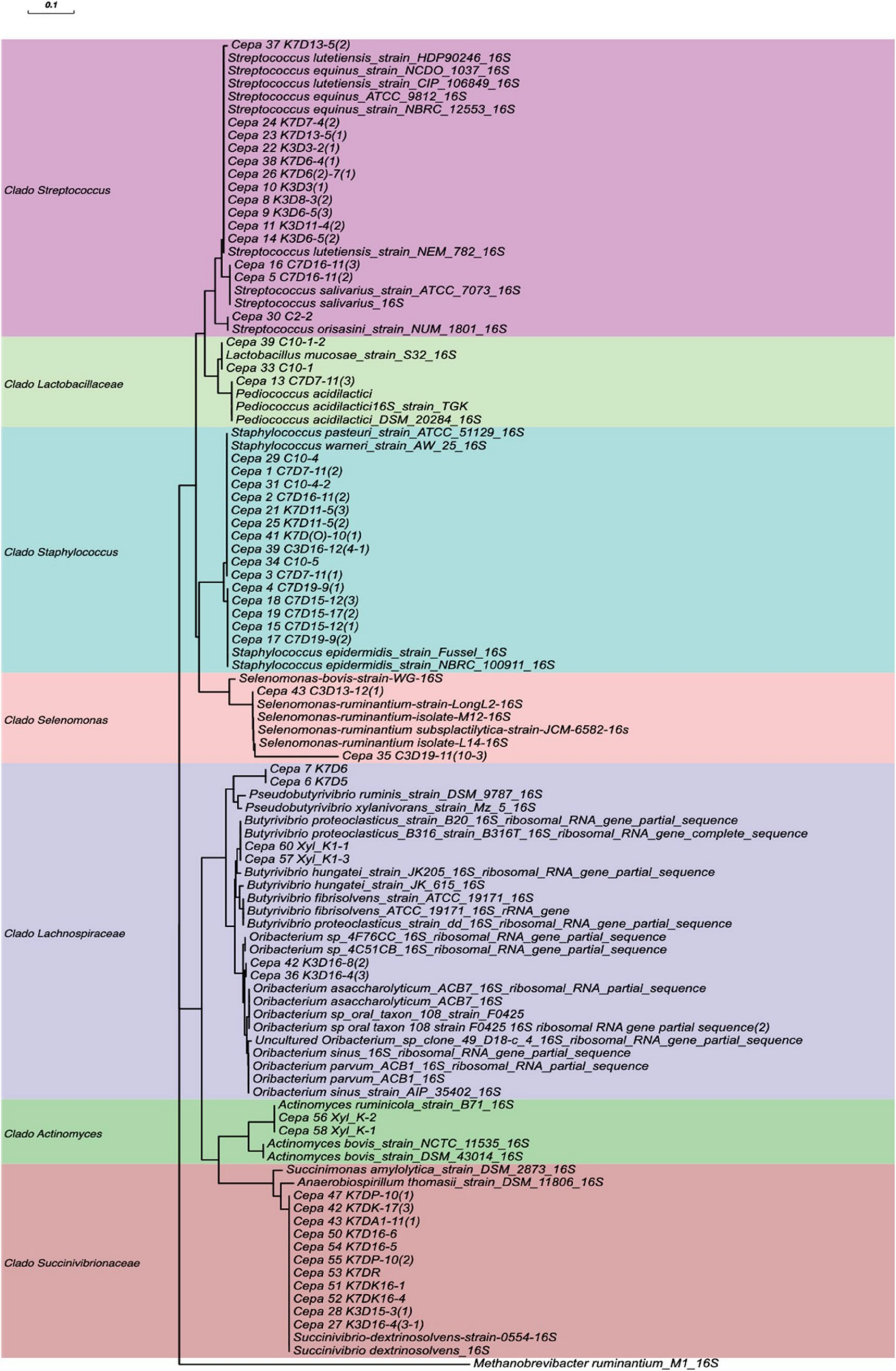
Phylogenetic dendrogram of isolates using the neighbor-joining method. The phylogenetic relationship among isolates is shown. The tree shows seven distinct clades. *Methanobrevibacter ruminantium* was used as the root for the tree. Numbers at the nodes indicate bootstrap values derived from the neighbor-joining analysis of 1000 replicates. The scale bar represents a 5% sequence divergence

The phylogenetic tree (Fig. 5) shows 7 clades that group the isolates with the reference sequences. The first clade corresponds to all the microorganisms of the genus *Streptococcus*; this clade was composed of 11 isolates (i37, i24, i23, i22, i38, i26, i10, i8, i9, i11, and i14). These were more closely related to the species *Streptococcus equinus* and *Streptococcus lutetiensis*. The second clade groups 3 isolates (i39, i33, and i13). These were associated with lactic acid bacteria from the family Lactobacillaceae with the genera *Limosilactobacillus* and *Pediococcus*. The third clade clusters 15 isolates related to the genus *Staphylococcus*; this clade was divided into 2 subclades the *S. warneri/pasteuri* and the *S. epidermidis* clade. The first subclade was more closely related to *S. warneri/pasteuri* subclade and included 5 isolates (i29, i31, i39, i34, and i3) obtained from the CAN medium, and 3 isolates (i41, i25, and i21), obtained from KNT. This second subclade was more closely related to *S. epidermidis* and included isolates (i4, i18, i19, i15, and i17) recovered from the CAN medium. The fourth clade contains *Selenomonas ruminantium*, with 2 isolates (i43 and i35) slightly divergent from the reference. The fifth clade includes 6 isolates, belonging to the family Lachnospiraceae, Pseudobutyrivibrio (i7 and i6), Butyrivibrio (i60 and i57), and Oribacterium (i42 and i36). These 6 isolates were recovered from the KNT medium at 3 and 7 days of incubation. The phylogenetic analysis of isolates i7 and i6 shows a relation to the *Pseudobutyrivibrio* genus, and these two isolates were related but divergent from the species *P. ruminis* and *P. xylanovorans*. Isolates i60 and i57 were related to the genus *Butyrivibrio.* However, the relation with the species assigned taxonomically (Table 1) was not observed in the phylogenetic analysis. Instead, the isolates are more related to the subclade of *B. proteoclasticus*. The isolates (i42 and i36) were more closely related to the genus *Oribacterium*; however, no close relation was observed to the reported species *O. assacharolitycum*, *O. sinus*, and *O. parvum* which suggests that these 2 isolates possibly represent not described species of the genus. These isolates were recovered from KNT after 3 days of incubation. The sixth and seventh clades include isolates i56 and i58, identified as *Actinomyces ruminicola*, and isolates i47, i42, i43, i50, i54, i55, i53, i51, i52, i28, and i27, identified as *Succinivibrio dextrinosolvens*, which are grouped with reference sequences for each species. These 13 isolates were obtained from the KNT medium at both incubation times, with most of the *S. dextrinosolvens* isolates recovered after 7 days of incubation.

### Comparison of Cultivable and Non-cultivable Diversity

The analysis of the 12 samples of both media used to isolate microorganisms yielded an average of 12,370 reads with a length of 300 base pairs and was classified into 166 operational taxonomic units (OTUs) assigned to 7 phyla. The results showed the persistence of the phyla Actinobacteria, Bacteroidetes, and Firmicutes in the CAN medium, with Firmicutes as the dominant phylum. Proteobacteria were not detected in the enrichment for this medium. In the KNT medium, Actinobacteria, Bacteroidetes, Firmicutes, and Proteobacteria were present, with Firmicutes showing the greatest increase in abundance. The phyla Euryarchaeota, Tenericutes, and Verrucomicrobia were also present in the KNT medium. Most of the 34 isolates recovered from the KNT medium, belonged to the Actinobacteria, Firmicutes, and Proteobacteria phyla. The genera *Olsenella*, *Prevotella*, *Selenomonas*, *Sharpea*, *Shuttleworthia*, and *Streptococcus* continued to be enriched by the CAN medium.

The results showed that most of the enriched genera over time were the same, with only 15% of the isolated genera being part of the single colony isolated fraction. A comparison of the libraries made over time and the relationship to the isolates for the KNT medium showed that the enriched genera differed from those observed in the first library.

**Fig. 6:**
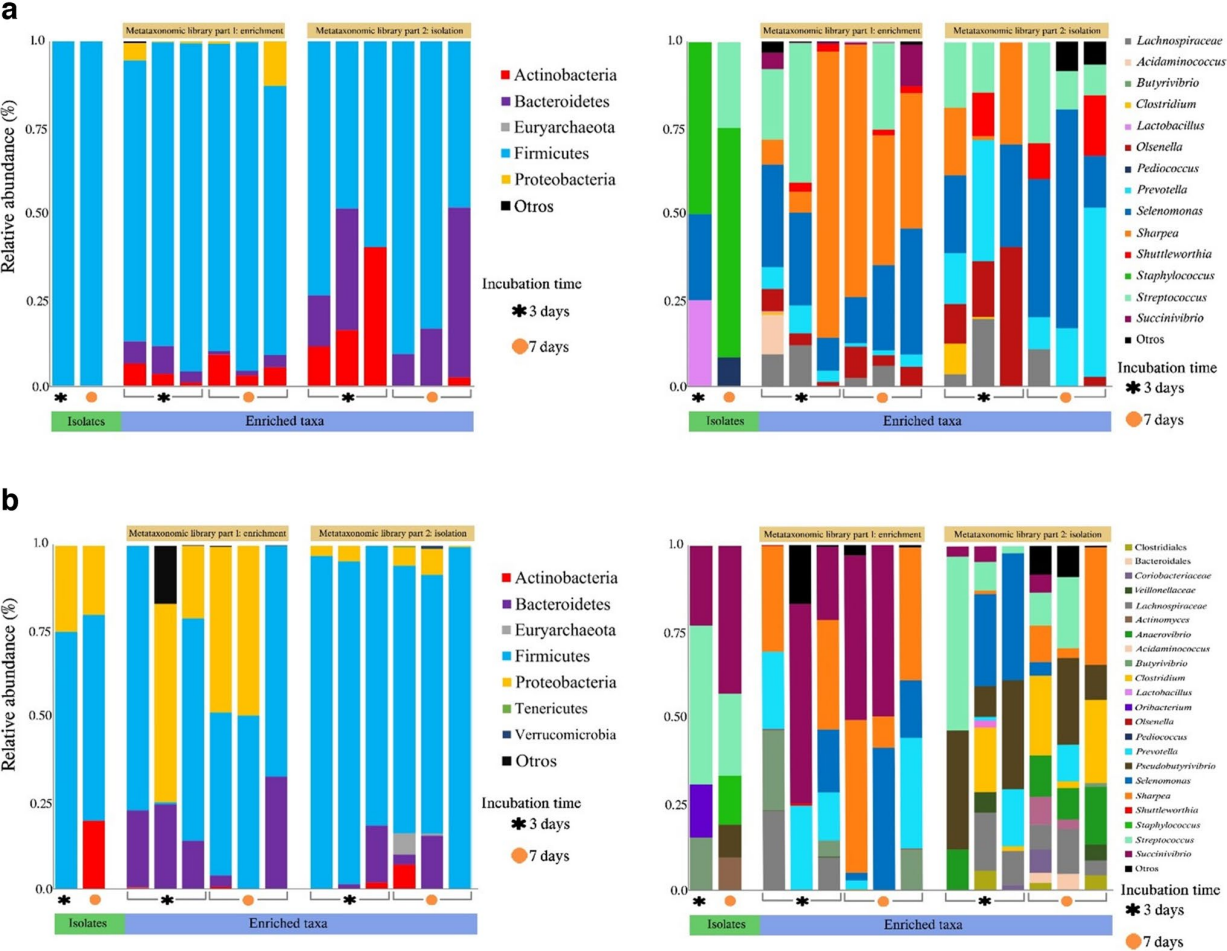
Comparative relative abundance of the microbial composition in the media and the obtained isolates. The bar graphs illustrate the relative abundance of phyla and genera of isolated microorganisms and juxtaposed with the diversity obtained by 16S rARN libraries for the first and second media evaluations. The comparison is conducted for sampling times 3 and 7 days using the 10^-12^ dilution. **a** The bar graphs are shown for CAN media. **b** The bar graphs are shown for KNT media

The observed variability in the enrichment of specific populations may be linked to differences in the ruminal fluid used in the medium, as it contains intrinsic nutrients that may not be removed during clarification and can provide growth factors that allow for a greater variety and richness of populations. The Lachnospiraceae family was observed in the medium with similar abundances over time (Fig. 6).

## Discussion

Culturing and isolating microbes present a significant challenge due to the complexities of replicating their natural environmental conditions. Despite the large number of bacteria within the rumen, only a tiny fraction of it has been isolated and described, leaving a significant portion of its bacterial community unidentified [38–40]. The research aimed to advance the isolation and characterization of diverse ruminal bacteria, seeking to uncover a broader spectrum of these organisms.

Our endeavors to accurately capture the taxonomic composition of the rumen faced particular challenges. The media assessed differed notably from actual ruminal content, with a pronounced dominance of the Bacteroidetes phyla exemplified by organisms related to the *Prevotella* genus. This contrasts with some studies that attribute a predominant presence of Firmicutes in rumen samples [41]. Interestingly, Bacteroidetes’ prominence is consistent across varied diets, both in the liquid and solid phase of the rumen, sometimes constituting up to 33% in the phyla [42, 43]. The significant number of OTUs with abundances below 1% suggests the presence of numerous unidentified or uncharacterized microorganisms. Such disparities might lead to broader taxonomic classifications, consolidating multiple organisms under larger groups such as families or orders and emphasizing the rumen’s rich bacterial diversity [40, 44–46].

In our methodological approach, we deployed eight distinct media and discerned a pronounced enrichment in Firmicutes and Proteobacteria. Most of the media enrich the same genus, though with varying abundances. Observations also indicated genera-specific preferences for certain media and conditions, with genera like *Shuttleworthia* and *Olsenella* showcasing specific media affinities [47–50]. The results showed that the closest dilutions to the initial sample maintained consistent diversity and richness. In contrast, after 10^−3^ dilution, there was a marked reduction in these metrics, and the community became more homogeneous at the highest dilution (10^−6^). The impact of dilutions on the microbial structural diversity in an ecosystem has been studied before, highlighting the nonlinear effects on diversity, richness, and homogeneity parameters [51]. The variations we observed might not only be due to dilution effects but could suggest stochastic processes, or “ecological drift.” This refers to changes in the abundance and identity of species within a microbial community over time, where community shifts occur due to random events such as birth, death, or reproduction. These changes can particularly impact rare microbial taxa [52, 53].

While the incubation duration did not significantly affect the community, previous studies have emphasized varied incubation periods (3, 8, 9 to 14 days) for bacterial colony isolation [30, 31]. Despite detailed incubation timelines, previous studies did not elucidate the outcomes associated with specific incubation durations. Considering the variations in media composition, our initial expectation was to selectively enrich and subsequently isolate a diverse range of microorganisms selectively. According to the literature [28–31], with the exception of media designed (GC, KYO, and TRB), each medium was anticipated to exhibit a distinct microbial enrichment profile. We expected an abundance of microorganisms such as *Pseudobutyrivibrio*, *Treponema*, *Actinomyces*, *Ruminococcus*, *Bacteroides*, *Eubacterium*, *Lachnospira*, *Micromonospora*, *Propionibacterium*, and *Coprococcus*. Contrary to expectations, despite varied media compositions, genera enrichment demonstrated consistency across media (Fig. 2), this observation can be related to a strong interaction between these genera, which can be enriched and prevail in different media. Notably, this research utilized molecular marker sequencing, which enables a more comprehensive examination of diversity composition independent of cultivation. However, the exploration comes with the caveat that it might amplify genes from metabolically inert or non-viable bacteria, an aspect not addressed in this study.

Our results indicate the presence of a core community of microorganisms in all eight media tested. The main disparities observed were related to the recovery of enriched taxa. To assess this, we employed two criteria: reproducibility during media preparation and the enrichment of a limited number of taxa. In our study, we recovered microorganisms such as *S. dextrinosolvens*, *S. lutetiensis*, *S. pasteuri*, *Pseudobutyrivibrio* sp., *Oribacterium* sp., *Butyrivibrio fibrisolvens*, and *Actinomyces ruminicola* from the KNT media used by [30]. In comparison, the authors isolated a total of 60 pure bacterial cultures from 1000 inoculated tubes, some of which were identified as belonging to the *genera Butyrivibrio*, *Oribacterium*, and *Pseudobutyrivibrio*, and were sourced from the rumen content of sheep. The KNT media were developed as a chemically defined medium designed to mimic the rumen environment while inhibiting the growth of mixed cultures. Despite this, mixed cultures were obtained in both our and the author’s studies. The comparison of metataxonomic results between both studies suggests differences in the enrichment of genera, which may be due to the use of ruminal fluid samples taken at different times. On the other hand, the enrichment and isolation of microorganisms using CAN media are considered the first report on the use of this media in literature. The consistency and reproducibility of the enriched taxa through time can be attributed to the composition of the media; CAN medium is considered as a chemically defined media. For both cases, the media showed a low percentage for recovering the taxa enriched; this suggests there are interactions between each taxon, having mutualistic interactions where two or more taxa grow together and are strongly dependent on each other. Also, there is the possibility of having unculturable taxa that compete with the culturable taxa, when both of them are cultivated in the roll tubes, the culturable one can survive first [54–57].

In conclusion, our study aimed to shed light on the challenges of culturing and isolating microorganisms from complex environments like the rumen. Despite using an extensive culture strategy (eight different media, three dilutions, and two incubation times), capturing a representative bacterial diversity from the rumen remains elusive. Our results showed a predominant enrichment of Firmicutes and Proteobacteria, which are relatively less prevalent in the rumen fluid. This alludes to the selective enrichment efficacy of our media for specific rumen microbiome groups. Variations in microbial composition can be attributed to the combined effects of media composition, dilution gradients, stochastic processes, and ecological drift. Our findings pave the way for future endeavors in rumen microbiology, emphasizing the need for refined methodologies and strategies to capture its microbial diversity holistically.

### Supplementary Information

ESM 1 (XLSX 30 kb)

ESM 2 (PNG 452 kb)

## Data Availability

All data are available under BioProjects PRJNA1004853, PRJNA956840, and OQ653374-OQ653426 (NCBI).
