# Supplementary figures and images for: Enhancing the Conventional Culture: the Evaluation of Several Culture Media and Growth Conditions Improves the Isolation of Ruminal Bacteria
Source: Microb Ecol. 2023 Dec 12;87(1):13. doi: 10.1007/s00248-023-02319-2 (PMC10713758; doi:10.1007/s00248-023-02319-2)

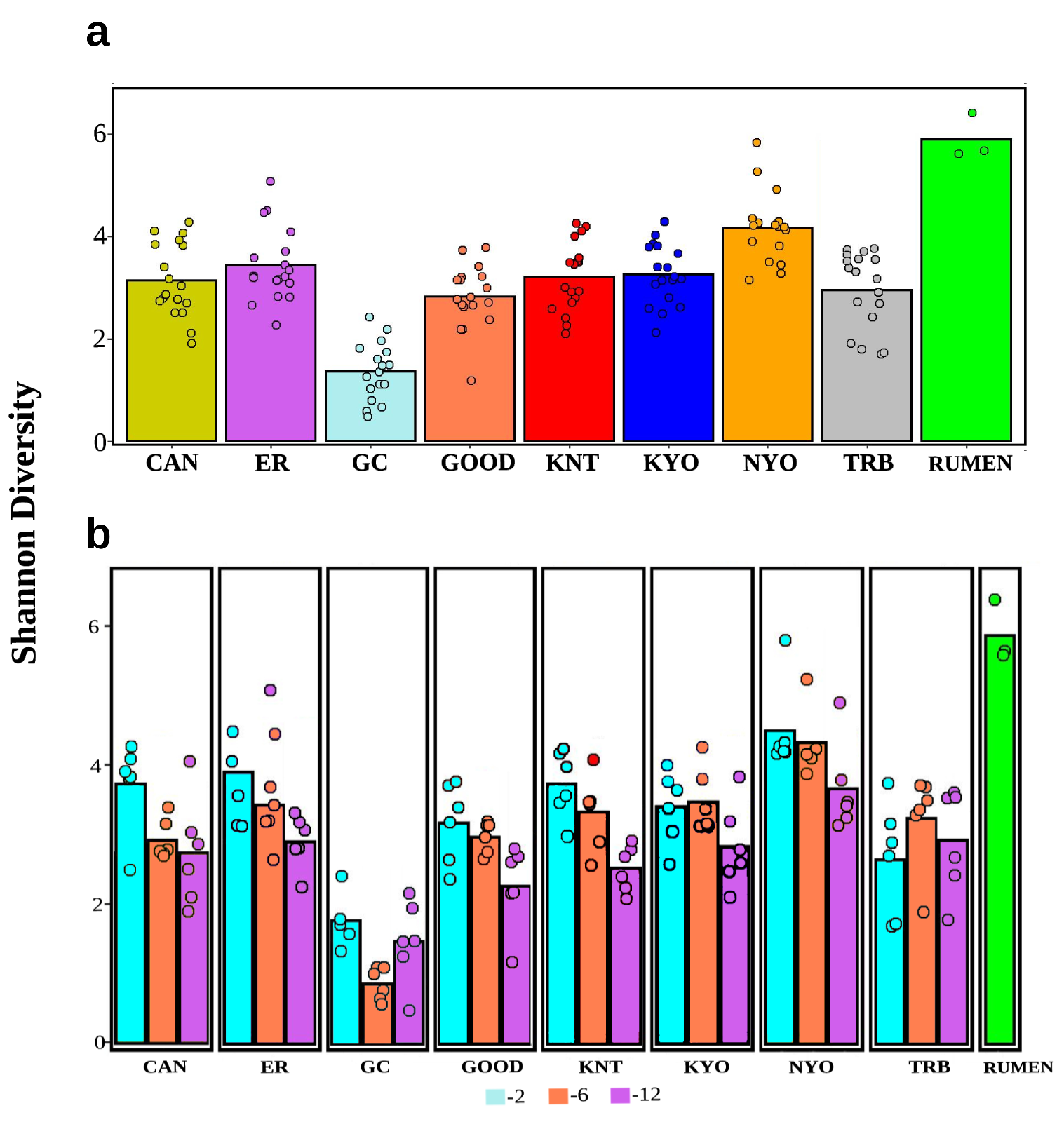

Supplement: Supplementary file 2 — (PNG 452 kb) [file 248_2023_2319_MOESM2_ESM.png]
